# 

**Published:** 1883-03

**Authors:** 


CONTENTS.

| | Page. |
|---|---|
| Rheumatism | 65 |
| Economy | 67 |
| Cold Feet | 68 |
| Laws of Health | 70 |
| Simple Remedies | 71 |
| Snake Bite—Rapid Cure by Phenic Acid. | 74 |
| Abscess of Antrum | 75 |
| Eucalyptus Trees in the Roman Campagna | 76 |
| Digestibility of Coffee and Sugar | 76 |
| In the Water—Instructions by Which we may Keep from Drowning | 77 |
| Cremation in Europe | 78 |
| How to Apply the Soda Remedy in Burns and Scalds | 79 |
| Analyses of Food | 81 |
| Dangers of Phosphorus | 83 |
| Who are the Gypsies ? | 84 |

